# Supplementary figures and images for: Reconstitution of a minimal ESX-5 type VII secretion system suggests a role for PPE proteins in the outer membrane transport of proteins
Source: mSphere. 2023 Sep 25;8(5):e00402-23. doi: 10.1128/msphere.00402-23 (PMC10597459; doi:10.1128/msphere.00402-23)

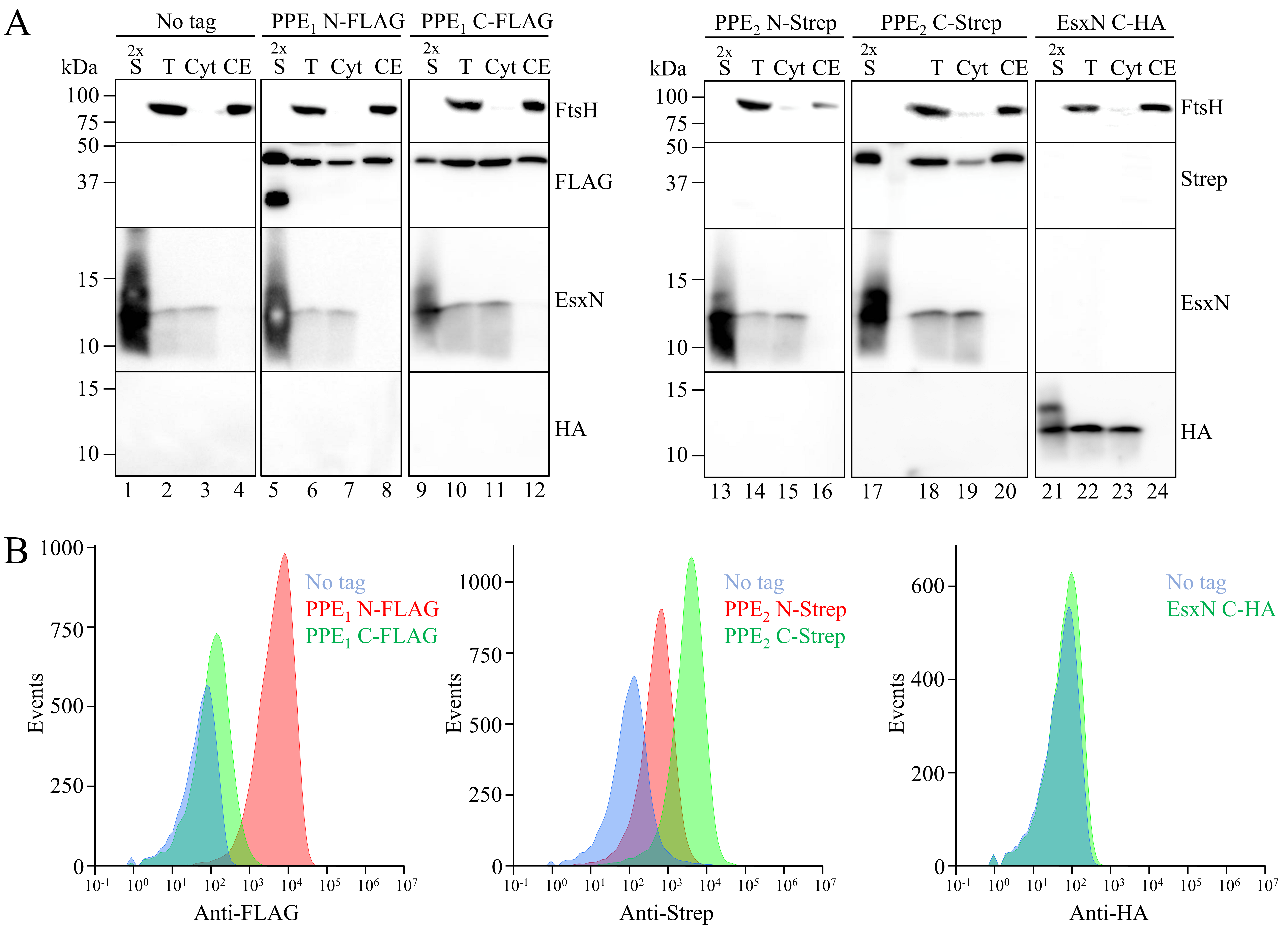

Supplement: Figure S1 — Subcellular fractionation of M. smegmatis carrying the esx-5Mxe plasmid expressing tagged ESX-5 substrates. [file msphere.00402-23-s0001.tif]

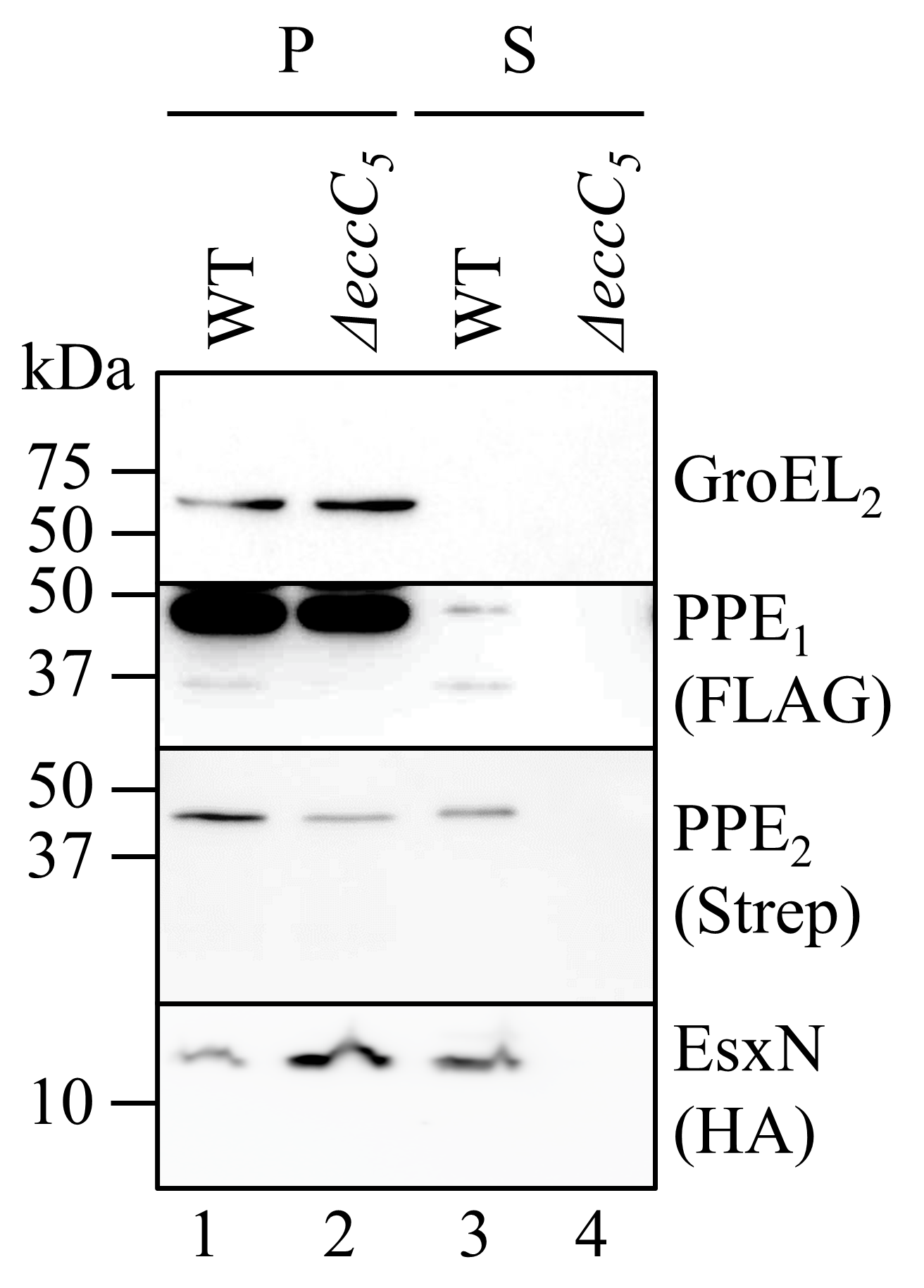

Supplement: Figure S2 — Secretion analysis of the esx-5-encoded PPE proteins by M. smegmatis carrying the esx-5Mxe ΔeccC5 plasmid. [file msphere.00402-23-s0002.tif]
