# Supplementary material for: Reconstitution of a minimal ESX-5 type VII secretion system suggests a role for PPE proteins in the outer membrane transport of proteins
Source: mSphere. 2023 Sep 25;8(5):e00402-23. doi: 10.1128/msphere.00402-23 (PMC10597459; doi:10.1128/msphere.00402-23)
Supplement: Supplemental Legends — Legends for Figures S1 and S2 and Tables S1, S2, and S3. [file msphere.00402-23-s0003.docx]

**Supplementary Figure 1. Subcellular fractionation of *M. smegmatis* carrying the *esx-5_Mxe_* plasmid expressing tagged ESX-5 substrates.**

(A) Cells expressing the *M. xenopi esx-5* locus encoding substrates with tags at the N or C- termini were grown in the presence of 0.05% Tween 80, after which supernatants (S) were separated from bacterial cells. The cells were subsequently fractionated into total (T), cytosol (Cyt) and cell envelope (CE) fractions. Fractions were loaded in a ratio of 2:1:1:1 for S:T:Cyt:CE. Antibody against FtsH (inner membrane protein) was used as fractionation control. (B) Surface exposure of substrates on whole cells was measured by flow cytometry of cells grown in the presence of Tween-80.

**Supplementary Figure 2. Secretion analysis of the *esx-5-*encoded PPE proteins by *M. smegmatis* carrying the *esx-5_Mxe_* Δ*eccC_5_* plasmid.** SDS-PAGE and immunoblot analysis of secreted fractions (S) and whole cell lysates (P) of *M. smegmatis* carrying the ESX-5_Mxe_ plasmid encoding the first PPE substrate (PPE_1_) with a FLAG-tag at the N-terminus, the second PPE protein (PPE_2_) with a Strep-tag at the C-terminus and EsxN with a HA-tag at the C-terminus and the same plasmid with *eccC_5_* deleted. Antibodies against GroEL2 were used as lysis control. Cells were grown in the presence of Tween-80.

**Table S1.** List of plasmids used in this study.

**Table S2.** List of oligonucleotides used in this study.

**Table S3****.** Overview of molecular cloning strategies for generating the plasmids shown in Table S1 and using primers shown in Table S2.
