# Supplementary material for: Reconstitution of a minimal ESX-5 type VII secretion system suggests a role for PPE proteins in the outer membrane transport of proteins
Source: mSphere. 2023 Sep 25;8(5):e00402-23. doi: 10.1128/msphere.00402-23 (PMC10597459; doi:10.1128/msphere.00402-23)
Supplement: Table S1 — Plasmids. [file msphere.00402-23-s0004.docx]

| **Nr.** | **Construct** | **Source** |
| --- | --- | --- |
| 1. | pMV *esx-5_Mxe_* | Beckham *et al*., 2017 |
| 2. | pMV *esx-5_Mxe_* ∆*eccB_5_* | this study |
| 3. | pMV *esx-5_Mxe_* ∆*eccC_5_* | this study |
| 4. | pMV *esx-5_Mxe_* ∆*pe/ppe* | this study |
| 5. | pMV *esx-5_Mxe_* ∆*esxM/N* | this study |
| 6. | pMV *esx-5_Mxe_* ∆*substrates* | this study |
| 7. | pMV *esx-5_Mxe_* ∆*espG_5_* | this study |
| 8. | pMV *esx-5_Mxe_* ∆*eccD_5_* | this study |
| 9. | pMV *esx-5_Mxe_* ∆*mycP_5_* | van Winden *et al*., 2020 |
| 10. | pMV *esx-5_Mxe_* ∆*eccE_5_* | this study |
| 11. | pMV *esx-5_Mxe_* ∆*eccA_5_* | this study |
| 12. | pMV *esx-5_Mxe_* membrane components (MC) | this study |
| 13. | pSMT3 *pe/ppe* whole | this study |
| 14. | pSMT3 *pe/ppe* ∆*pe/ppe1* | this study |
| 15. | pSMT3 *pe/ppe* ∆methyltransferase | this study |
| 16. | pSMT3 *pe/ppe* ∆*pe/ppe2* | this study |
| 17. | pMV *esx-5_Mxe_* *ppe1* FLAG N-term | this study |
| 18. | pMV *esx-5_Mxe_* *ppe1* FLAG C-term | this study |
| 19. | pMV *esx-5_Mxe_* *ppe2* Strep N-term | this study |
| 20. | pMV *esx-5_Mxe_* *ppe2* Strep C-term | this study |
| 21. | pMV *esx-5_Mxe_* *esxN-HA* | this study |
| 22. | pMV *esx-5_Mxe_* *ppe1* FLAG N-term, *ppe2* Strep C-term, *esxN-HA*, No Strep on *eccC_5_* | this study |
| 23. | pMV *esx-5_Mxe_* *ppe1* FLAG N-term, *ppe2* Strep C-term, *esxN-HA*, ∆*eccC_5_* | this study |

**Table S1**: List of plasmids used in this study.
