# Supplementary material for: Reconstitution of a minimal ESX-5 type VII secretion system suggests a role for PPE proteins in the outer membrane transport of proteins
Source: mSphere. 2023 Sep 25;8(5):e00402-23. doi: 10.1128/msphere.00402-23 (PMC10597459; doi:10.1128/msphere.00402-23)
Supplement: Table S2 — Oligonucleotides. [file msphere.00402-23-s0005.docx]

**Table S2**: List of oligonucleotides used in this study.

| **Nr.** | **Primer name** | **Primer sequence 3' -5'** |
| --- | --- | --- |
| 1. | ESX-5 xen del.EccB 1 FW | TGTTTGCTTGTCGGACACCT |
| 2. | ESX-5 xen del.EccB 1 RV | CTATTTGAGTCACGATCCCCGTTC |
| 3. | ESX-5 xen del.EccB 2 FW | GATCGTGACTCAAATAGGCACCATGGGTGGCACTG |
| 4. | ESX-5 xen del.EccB 2 RV | TCCCATTGCGGGTCTTCGAT |
| 5. | ESX-5 xen del.EccC FW | GTACGCGAAGAACCACTTAATTAATGC |
| 6. | ESX-5 xen del.EccC RV | GAGGTGCCTGCCTTAAGTCACTTCGGTACCGCCAATTC |
| 7. | Del EspG xen FW | GGCGGGGGCTTCTCTAGATG |
| 8. | Del EsxM/N xen 1 rv | TGCGGTTGGTTGTTACTGAATATCTCCTTAGCTCGAAATCGGCTG |
| 9. | Del EsxM/N xen 2 fw | TAACAACCAACCGCAGGCGC |
| 10. | Del EsxM/N xen 2 rv | CATCTCGACCGAATACGTAAGTGAAAG |
| 11. | Xen ESX-5 del sub. INF. FW | CGAGAAGTAAAGCTCTTAAGTAACAACCAACCGCAGGCGCG |
| 12. | Del EspG xen FW | GGCGGGGGCTTCTCTAGATG |
| 13. | Del EspG xen RV | CATCTCGACCGAATACgtaGGCTGCTCCCATGTTGGCGTG |
| 14. | Del EccE clean 1 FW | GATCCTGCGCGGCCGCTCAT |
| 15. | Del EccE clean 1 RV | CAGCCGTGGGCGTCGTCACGTCATGGCTGCCTCCCTTGTCG |
| 16. | Del EccE clean 2 FW | CGTGACGACGCCCACGGCTGGTG |
| 17. | Del EccE clean 2 RV | CGTCGACATCGATAAGCTTTGG |
| 18. | Del EccA xen FW | GCCATCTGCCGGACGCGTG |
| 19. | Del EccA xen RV | GTCGACATCGATAAGCTTCATCGGGCCGCCGCCGGG |
| 20. | Xen whole PE/PPE inf FW | GAGGAATCACGCTAGCGATGTCGTTCGTGACC |
| 21. | Xen whole PE/PPE inf RV | AGATATCCATGGATCCTAACCGGCGTAGACC |
| 22. | Xen tag substr 1 FW | CGAGAAGTAAAGCTCTTAAGGCAGGCAC |
| 23. | Xen 1^st^ PPE flag C 1 RV | TGTCGTCATCGTCTTTGTAGTCGCCGGCGAACGGTGGGCGTG |
| 24. | Xen 1^st^ PPE Flag C 2 FW | ACAAAGACGATGACGACAAGTAGCAACACGCGAAGCGCCG |
| 25. | Xen tag substr 2 RV | TGCATCTCGACCGAATACGTAAGTGAAA |
| 26. | Xen 2^nd^ PPE Strep C 1 RV | TTCAAATTGGGGATGCGACCATGATCCTCCACCGGCGTAGACCGGGCG |
| 27. | Xen 2^nd^ PPE Strep C 2 FW | TCGCATCCCCAATTTGAAAAATAGGAAAGGGGGCCAGCCG |
| 28. | Xen EsxN-HA 1 RV | TAATCAGGAACATCATACGGATAGGCCCAGCTGGAGCCGAC |
| 29. | Xen EsxN-HA 2 FW | CGTATGATGTTCCTGATTATGCTTAACAACCAACCGCAGGCGC |
| 30. | Xen 1^st^ PPE flag Nterm 1 RV | CGAACCTCCCGAACCTCCCTTGTCGTCATCGTCTTTGTAGTCCATTAGTACTCTCCTTAGCCCGCCG |
| 31. | Xen 1^st^ PPE Flag Nterm 2 FW | GGAGGTTCGGGAGGTTCGGTGCTGGACTTCGGGGCATTTC |
| 32. | Xen 2^nd^ PPE Strep Nterm 1 RV | CGAACCTCCCGAACCTCCTTTTTCAAATTGGGGATGCGACCACATCTAGAGAAGCCCCCGCC |
| 33. | Xen 2^nd^ PPE Strep Nterm 2 FW | GGAGGTTCGGGAGGTTCGTTGGATTTCGGGCTGTTTCCG |
| 34. | Rem Str EccC5xen FW | CCGACCTGCGCCGGTTTAAATAGCTTAAGGCAGGCACCTCTTGAG |
| 35. | Rem Str EccC5xen RV | CTCAAGAGGTGCCTGCCTTAAGCTATTTAAACCGGCGCAGGTCGG |
