# Supplementary material for: Reconstitution of a minimal ESX-5 type VII secretion system suggests a role for PPE proteins in the outer membrane transport of proteins
Source: mSphere. 2023 Sep 25;8(5):e00402-23. doi: 10.1128/msphere.00402-23 (PMC10597459; doi:10.1128/msphere.00402-23)
Supplement: Table S3 — Overview of molecular cloning strategies for generating the plasmids shown in Table S1 and using primers shown in Table S2. [file msphere.00402-23-s0006.docx]

**Table S3:** Overview of molecular cloning strategies for generating the plasmids shown in Table S1 and using primers shown in Table S2.

| **Nr.** | **Construct** | **Starting backbone** | **Restriction sites used to cut the backbone** | **Primers used** | **PCR product size** | **Comments** |
| --- | --- | --- | --- | --- | --- | --- |
| 2. | pMV *esx-5_Mxe_* ∆*eccB_5_* | pMV *esx-5_Mxe_* | EcoRV, SexAI | 1, 2 | 330 bp |  |
|  |  |  |  | 3, 4 | 1140 bp | Restricted backbone and the two PCR products were ligated with InFusion HD. |
| 3. | pMV *esx-5_Mxe_* ∆*eccC_5_* | pMV *esx-5_Mxe_* | PacI, AflII | 5, 6 | 2174 bp | Restricted backbone and the PCR product were ligated with InFusion HD. |
| 4. | pMV *esx-5_Mxe_* ∆*pe/ppe* | pMV *esx-5_Mxe_* | AflII, XbaI |  |  | Restriction removes *pe/ppe* genes. Resulting backbone was recircularized. |
| 5. | pMV *esx-5_Mxe_* ∆*esxM/N* | pMV *esx-5_Mxe_* | Xbai, SnaBI | 7, 8 | 1356 bp | Restricted backbone and the two PCR products were ligated in InFusion HD. |
|  |  |  |  | 9, 10 | 1061 bp |  |
| 6. | pMV *esx-5_Mxe_* ∆*substr.* | pMV *esx-5_Mxe_* | AflII, SnaBI | 11, 10 | 1082 bp | Restricted backbone and the PCR product were ligated with InFusion HD. |
| 7. | pMV *esx-5_Mxe_* ∆*espG_5_* | pMV *esx-5_Mxe_* | XbaI, SnaBI | 12, 13 | 2053 bp | Restricted backbone and the PCR product were ligated with InFusion HD. |
| 8. | pMV ESX-5xen ∆*eccD_5_* | pMV *esx-5_Mxe_* | SnaBI, NdeI |  |  | Restriction removes *eccD_5_.* Resulting backbone was recircularized. |
| 10. | pMV ESX-5xen ∆*eccE_5_* | pMV *esx-5_Mxe_* | NdeI, HindIII | 14, 15 | 2068 bp | Restricted backbone and the two PCR products were ligated with InFusion HD. |
|  |  |  |  | 16, 17 | 2607 bp |  |
| 11. | pMV ESX-5xen ∆EccA5 | pMV *esx-5_Mxe_* | MluI, HindIII | 18, 19 | 1129 bp | Restricted backbone and the PCR product were ligated with InFusion HD. |
| 12. | pMV ESX-5xen MC | pMV *esx-5_Mxe_* ∆EccA5 | AflII, SnaBI |  |  | Restriction removes all substrate genes and *espG_5_.* Resulting backbone was recircularized. |
| 13. | pSMT3 *pe/ppe* whole | pSMT3 empty |  | 20, 21 | 4583 bp | Backbone and PCR product were ligated with InfusionHD. |
| 14. | pSMT3 pe/ppe ∆*pe/ppe1* | pSMT3 *pe/ppe* whole | NheI |  |  | Restriction removes 1st *pe/ppe* genes. Resulting backbone was recircularized. |
| 15. | pSMT3 *pe/ppe*∆methyltransf. | pSMT3 *pe/ppe* whole | NheI, BamHI |  |  | Partial restriction to delete methyltransferase. Resulting backbone was recircularized. |
| 16. | pSMT3 *pe/ppe* ∆*pe/ppe2* | pSMT3 *pe/ppe* whole | BamHI |  |  | Restriction remove 2nd *pe/ppe* genes. Resulting backbone was recircularized. |
| 17. | pMV *esx-5_Mxe_* *ppe1* FLAG N-term | pMV *esx-5_Mxe_* | AflII, SnaBI | 22, 30 | 688 bp | Restricted backbone and the two PCR products were ligated with InFusion HD. |
|  |  |  |  | 31, 25 | 6035 bp |  |
| 18. | pMV *esx-5_Mxe_* *ppe1* FLAG C-term | pMV *esx-5_Mxe_* | AflII, SnaBI | 22, 23 | 1934 bp | Restricted backbone and the two PCR products were ligated with InFusion HD. |
|  |  |  |  | 24, 25 | 4769 bp |  |
| 19. | pMV *esx-5_Mxe_* *ppe2* Strep N-term | pMV *esx-5_Mxe_* | AflII, SnaBI | 22, 32 | 3716 bp | Restricted backbone and the two PCR products were ligated with InFusion HD. |
|  |  |  |  | 33, 25 | 3005 bp |  |
| 20. | pMV *esx-5_Mxe_* *ppe2* Strep C-term | pMV *esx-5_Mxe_* | AflII, SnaBI | 22, 26 | 4910 bp | Restricted backbone and the two PCR products were ligated with InFusion HD. |
|  |  |  |  | 27, 25 | 1802 bp |  |
| 21. | pMV *esx-5_Mxe_* *esxN-HA* | pMV *esx-5_Mxe_* | AflII, SnaBI | 22, 28 | 5621 bp | Restricted backbone and the two PCR products were ligated with InFusion HD. |
|  |  |  |  | 29, 25 | 1802 bp |  |
| 22. | pMV *esx-5_Mxe_* *ppe1* FLAG N-term, *ppe2* Strep C-term, *esxN-HA*, no Strep on *eccC_5_.* | pMV *esx-5_Mxe_* *ppe1* Flag N-term | AflII, SnaBI | 22, 26 | 4910 bp | Restricted backbone and the two PCR products were ligated with InFusion HD into an intermediate plasmid. |
|  |  | pMV *esx-5_Mxe_* *esxN-HA* | AflII, SnaBI | 27, 25 | 1802 bp |  |
|  |  | Intermediate plasmid | DraI, AflII | 34, 35 |  | Restricted intermediate plasmid was religated with InFusion HD with the overlapping primers to swap the TwinStrep at the C-terminus of *eccC_5_* with a stop codon. |
| 23. | pMV *esx-5_Mxe_* *ppe1* FLAG N-term, *ppe2* Strep C-term, *esxN-HA*, ∆*eccC_5_* | pMV *esx-5_Mxe_* *ppe1* FLAG N-term, *ppe2* Strep C-term, *esxN-HA*, No Strep on *eccC_5_.* | PacI, AflII | 5, 6 | 2174 bp | Restricted backbone and the PCR product were ligated with InFusion HD. |
